# Supplementary material for: Targeted next-generation sequencing for comprehensive diagnosis and drug resistance detection in pulmonary and extrapulmonary tuberculosis: a single-center retrospective study
Source: Microbiol Spectr. 2026 Feb 27;14(4):e01698-25. doi: 10.1128/spectrum.01698-25 (PMC13055381; doi:10.1128/spectrum.01698-25)
Supplement: Supplemental material — Supplemental methods; Fig. S1 and S2; Tables S1 to S6. [file spectrum.01698-25-s0001.docx]

**Targeted Next-Generation Sequencing for Comprehensive Diagnosis and Drug Resistance Detection in Pulmonary and Extrapulmonary Tuberculosis: A Single-Center Retrospective Study**

**Supplementary methods**

**tNGS assay**

In our investigation, we employed a multiplex-PCR based tNGS assay to analyze biological specimens from patients suspected of having TB. We collected specimens from these individuals, ensuring that at least 800 μL of each specimen was available for analysis. The microbial DNA was extracted using the GenK© magnetic bead-based DNA/RNA extraction kit designed for nucleic acid extraction and purification. For the detection of TB pathogens and their resistance mutations, the extracted DNA was amplified using targeted multiplex-PCR with a custom primer panel. This panel targeted specific regions of 64 microbial species and 158 drug resistance sites associated with M. tuberculosis, utilizing the GenK© tuberculosis infection pathogen nucleic acid detection kit. Following the amplification, adapters were added to the enriched DNA fragments to facilitate library construction. The libraries were then sequenced on the MGISEQ-2000 platform, producing 50 bp single-end reads.

The sequencing output underwent an initial quality control check with fastp(v0.23.2), where we ensured that each sample produced at least 1 million clean reads with more than 85% achieving a Q30 quality score.^1^ Post-QC, we removed host-derived reads by aligning the data against the human T2T reference genome CHM13V2.0 using bowtie2(v2.3.5.1).2 This step was crucial to purify our dataset for subsequent pathogen-specific analysis. The pathogen-specific reads were then aligned to the amplicon library of our primer panel using bwa-mem (v0.7.1), allowing precise identification of pathogen sequences.^3^ A key component of our analysis was the detection of drug resistance mutations in the TB pathogens, which we performed by referencing the standard *M. tuberculosis* strain H37Rv. This was achieved using the mpileup method in samtools to identify and analyze mutations.^4^ We comprehensively summarized and statistically analyzed the data, which enabled us to construct a detailed profile of the pathogens present in the samples. We set specific threshold values for pathogen detection and drug resistance prediction, culminating in a report that provides clear and actionable insights for clinical interpretation.

**Supplementary figures**

**
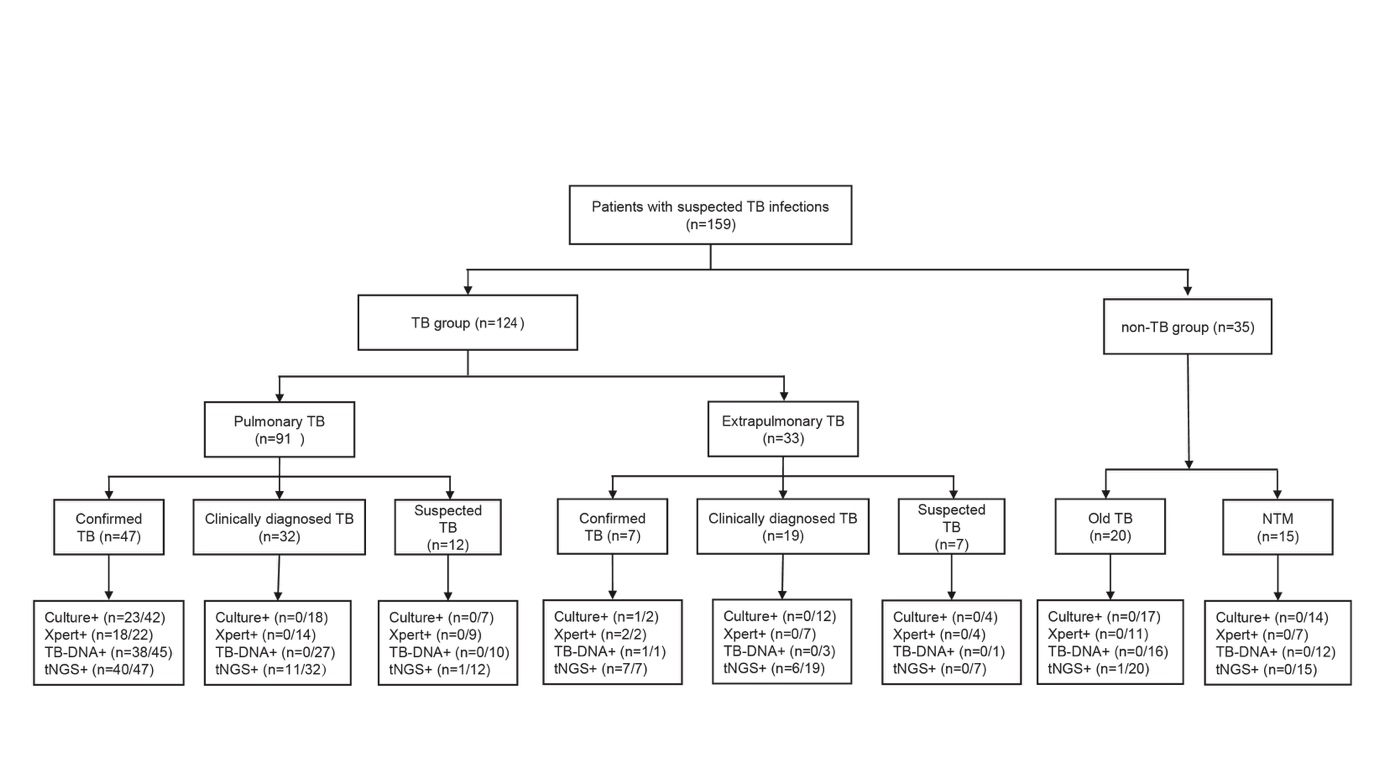
**

**Figure S1** Grouping of Enrolled Patients and Positive Detection Rates for MTBC (TB) by Different Diagnostic Methods.

**
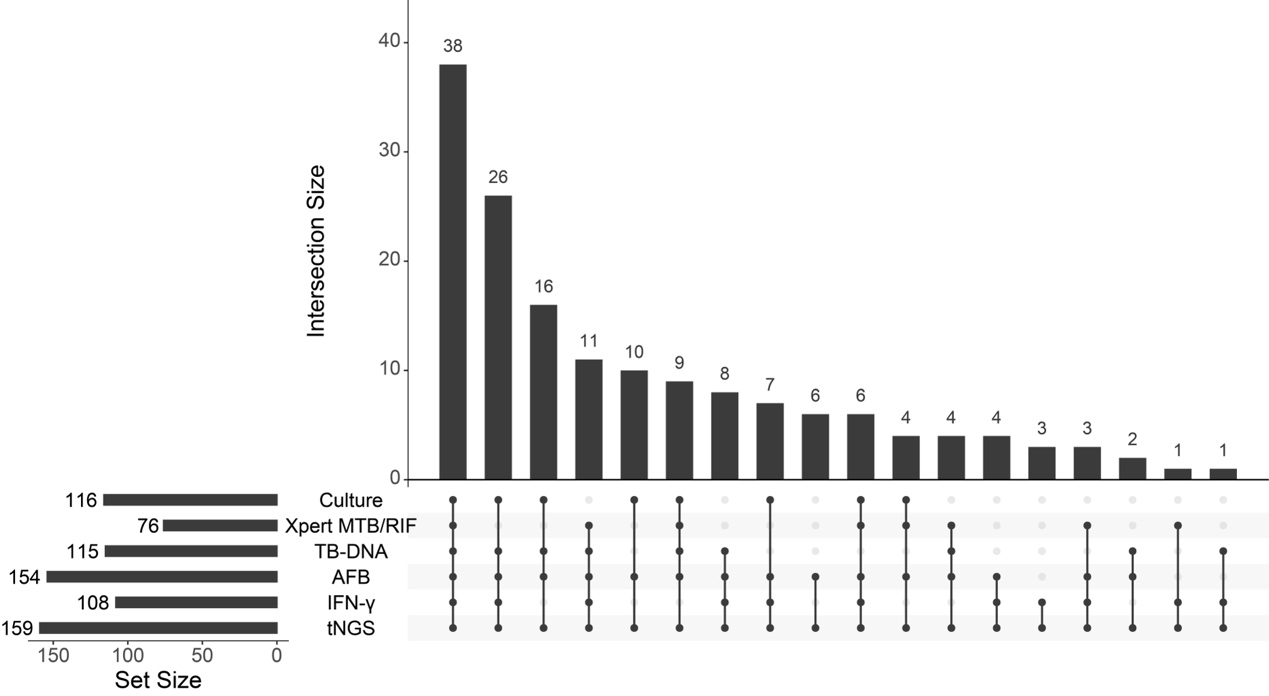
**

**Figure S2** Overlap of Sample Coverage Across Different Diagnostic Methods

**Supplementary tables**

**Table S1** Counts of Each Sample Type in The Study

| **Sample type** | **N** | **%** |
| --- | --- | --- |
| BALF | 85 | 53.50% |
| Sputum | 40 | 25.20% |
| Pus | 20 | 12.60% |
| Cerebrospinal fluid (CSF) | 10 | 6.30% |
| Pleural fluid | 2 | 1.30% |
| Lung tissue | 1 | 0.60% |
| Peritoneal fluid | 1 | 0.60% |

**Table S2** Consistency of TB Detection Results with Culture for Four Diagnostic Methods

| **Group** | **Method** | **N** | **PPA^a^** | **NPA** | **OPA** |
| --- | --- | --- | --- | --- | --- |
| **All samples** | Xpert | 57 | 100.0% (8/8) | 81.6% (40/49) | 84.2% (48/57) |
|  | TB-DNA | 89 | 76.2% (16/21) | 72.1% (49/68) | 73.0% (65/89) |
|  | AFB | 116 | 54.2% (13/24) | 88.0% (81/92) | 81.0% (94/116) |
|  | tNGS | 116 | 91.7% (22/24) | 73.9% (68/92) | 77.6% (90/116) |
|  |  |  |  |  |  |
| **PTB** | Xpert | 48 | 100.0% (8/8) | 77.5% (31/40) | 81.3% (39/48) |
|  | TB-DNA | 85 | 76.2% (16/21) | 71.9% (46/64) | 72.9% (62/85) |
|  | AFB | 98 | 56.5% (13/23) | 85.3% (64/75) | 78.6% (77/98) |
|  | tNGS | 98 | 91.3% (21/23) | 73.3% (55/75) | 77.6% (76/98) |
|  |  |  |  |  |  |
| **EPTB** | Xpert | 9 | - | 100.0% (9/9) | 100.0% (9/9) |
|  | TB-DNA | 4 | - | 75.0% (3/4) | 75.0% (3/4) |
|  | AFB | 18 | 0.0% (0/1) | 100.0% (17/17) | 94.4% (17/18) |
|  | tNGS | 18 | 100.0% (1/1) | 76.5% (13/17) | 77.8% (14/18) |

1. PPA: Positive percent agreement, NPA: Negative percent agreement, OPA: Overall percent agreement

**Table S3** Consistency of TB Detection Results Among tNGS, Xpert, and TB-DNA

| **Group** | **Method** | **N** | **PPA** | **NPA** | **OPA** | **PPV** | **NPV** | **p-value** |
| --- | --- | --- | --- | --- | --- | --- | --- | --- |
| **All samples** | tNGS vs Xpert | 76 | 90.0% (18/20) | 85.7% (48/56) | 86.8% (66/76) | 69.2% | 96.0% | 0.109 |
|  | tNGS vs TB-DNA | 115 | 87.2% (34/39) | 84.2% (64/76) | 85.2% (98/115) | 73.9% | 92.8% | 0.143 |
|  | TB-DNA vs Xpert | 62 | 77.8% (14/18) | 90.9% (40/44) | 87.1% (54/62) | 77.8% | 90.9% | 1.000 |
| **PTB** | tNGS vs Xpert | 63 | 88.9% (16/18) | 86.7% (39/45) | 87.3% (55/63) | 72.7% | 95.1% | 0.289 |
|  | tNGS vs TB-DNA | 110 | 86.8% (33/38) | 84.7% (61/72) | 85.5% (94/110) | 75.0% | 92.4% | 0.210 |
|  | TB-DNA vs Xpert | 60 | 77.8% (14/18) | 90.5% (38/42) | 86.7% (52/60) | 77.8% | 90.5% | 1.000 |
| **EPTB** | tNGS vs Xpert | 13 | 100.0% (2/2) | 81.8% (9/11) | 84.6% (11/13) | 50.0% | 100.0% | 0.500 |
|  | tNGS vs TB-DNA | 5 | 100.0% (1/1) | 75.0% (3/4) | 80.0% (4/5) | 50.0% | 100.0% | 1.000 |
|  | TB-DNA vs Xpert | 2 | - | 100.0% (2/2) | 100% (2/2) |  |  |  |

*p-values were calculated using McNemar’s test to assess statistical differences in paired proportions between the compared methods.

**Table S4** Clinical Diagnostic Performance of Various TB Pathogen Detection Methods

| **Group** | **Method** | **N** | **TP^a^** | **FN** | **FP** | **TN** | **Sensitivity** | **Specificity** | **Accuracy** |
| --- | --- | --- | --- | --- | --- | --- | --- | --- | --- |
| **All sample** | Culture | 116 | 24 | 61 | 0 | 31 | 28.2% | 100.0% | 47.4% |
|  | Xpert | 76 | 20 | 38 | 0 | 18 | 34.5% | 100.0% | 50.0% |
|  | TB-DNA | 115 | 39 | 48 | 0 | 28 | 44.8% | 100.0% | 58.3% |
|  | AFB | 154 | 19 | 100 | 9 | 26 | 16.0% | 74.3% | 29.2% |
|  | γ-IFN | 108 | 57 | 27 | 13 | 11 | 67.9% | 45.8% | 63.0% |
|  | tNGS | 159 | 65 | 59 | 1 | 34 | 52.4% | 97.1% | 62.3% |
|  |  |  |  |  |  |  |  |  |  |
| **PTB** | Culture | 98 | 23 | 44 | 0 | 31 | 34.3% | 100.0% | 55.1% |
|  | Xpert | 63 | 18 | 27 | 0 | 18 | 40.0% | 100.0% | 57.1% |
|  | TB-DNA | 110 | 38 | 44 | 0 | 28 | 46.3% | 100.0% | 60.0% |
|  | AFB | 125 | 17 | 73 | 9 | 26 | 18.9% | 74.3% | 34.4% |
|  | IFN-γ | 88 | 43 | 21 | 13 | 11 | 67.2% | 45.8% | 61.4% |
|  | tNGS | 126 | 52 | 39 | 1 | 34 | 57.1% | 97.1% | 68.3% |
|  |  |  |  |  |  |  |  |  |  |
| **EPTB** | Culture | 18 | 1 | 17 | 0 | 0 | 5.6% | - | 5.6% |
|  | Xpert | 13 | 2 | 11 | 0 | 0 | 15.4% | - | 15.4% |
|  | TB-DNA | 5 | 1 | 4 | 0 | 0 | 20.0% | - | 20.0% |
|  | AFB | 29 | 2 | 27 | 0 | 0 | 6.9% | - | 6.9% |
|  | IFN-γ | 20 | 14 | 6 | 0 | 0 | 70.0% | - | 70.0% |
|  | tNGS | 33 | 13 | 20 | 0 | 0 | 39.4% | - | 39.4% |

1. TP：True positive, FN: False negative, FP: False positive, TN: True negative

**Table S5** Frequency and Detection Rate of Various NTM Subtypes Identified by tNGS Across All Samples

|  | tNGS | N=159 |
| --- | --- | --- |
|  | n | % |
| MAC^a^ | 13 | 8.20% |
| *M. chelonei* | 5 | 3.10% |
| *M. abscessus* | 2 | 1.30% |
| *M. kansasii^b^* | 1 | 0.60% |
| Total | 21 |  |

a. MAC: Mycobacterium avium complex

b. Co-detected with M. chelonei

**Table S6** Diagnostic Scope of Pathogens and Drug Resistance Mutations by tNGS

| **Pathongens** |  |  |
| --- | --- | --- |
| **Type of pathognes** | **Name of pathogens** |  |
| mycobacteria | *Mycobacterium tuberculosis complex* |  |
| mycobacteria | *Mycobacteroides abscessus* |  |
| mycobacteria | *Mycobacterium avium complex MAC* |  |
| mycobacteria | *Mycobacterium kansasii* |  |
| mycobacteria | *Mycobacteroides chelonae* |  |
| mycobacteria | *Mycolicibacterium fortuitum* |  |
| mycobacteria | *Mycobacterium marinum* |  |
| mycobacteria | *Mycobacterium haemophilum* |  |
| mycobacteria | *Mycobacterium gordonae* |  |
| mycobacteria | *Mycobacterium xenopi* |  |
| mycobacteria | *Mycolicibacterium vaccae* |  |
| mycobacteria | *Mycobacterium malmoense* |  |
| mycobacteria | *Mycobacterium scrofulaceum* |  |
| mycobacteria | *Mycobacterium ulcerans* |  |
| mycobacteria | *Mycobacteroides abscessus subsp. massiliense* |  |
| mycobacteria | *Mycobacteroides abscessus subsp. bolletii* |  |
| mycobacteria | *Mycobacterium simiae* |  |
| bacteria G+ | *Streptococcus pneumoniae* |  |
| bacteria G+ | *Staphylococcus aureus* |  |
| bacteria G- | *Acinetobacter baumannii* |  |
| bacteria G+ | *Nocardia farcinica* |  |
| bacteria G+ | *Nocardia cyriacigeorgica* |  |
| bacteria G+ | *Nocardia brasiliensis* |  |
| bacteria G+ | *Nocardia asteroids* |  |
| bacteria G+ | *Nocardia abscessus* |  |
| bacteria G- | *Burkholderia mallei* |  |
| bacteria G- | *Haemophilus influenzae* |  |
| bacteria G- | *Klebsiella aerogenes* |  |
| bacteria G- | *Proteus mirabilis* |  |
| bacteria G- | *Enterobacter cloacae complex* |  |
| bacteria G- | *Klebsiella pnenmoniae* |  |
| bacteria G- | *Pseudomonas aeruginosa* |  |
| bacteria G- | *Stenotrophomonas maltophilia* |  |
| bacteria G- | *Escherichia coli* |  |
| bacteria G- | *Legionella pneumophila* |  |
| bacteria G+ | *Tropheryma whipplei* |  |
| bacteria G- | *Citrobacter freundii* |  |
| bacteria G- | *Serratia marcescens* |  |
| fungi | *Aspergillus* |  |
| fungi | *Candida albicans* |  |
| fungi | *Candida tropicalis* |  |
| fungi | *Candida glabrata* |  |
| fungi | *Candida krusei* |  |
| fungi | *Aspergillus fumigatus* |  |
| fungi | *Aspergillus flavus* |  |
| fungi | *Aspergillus terreus* |  |
| fungi | *Aspergillus niger* |  |
| fungi | *Pneumocystis jirovecii* |  |
| fungi | *Cryptococcus neoformans* |  |
| DNA virus | *Human adenovirus B* |  |
| DNA virus | *Human adenovirus C* |  |
| DNA virus | *Human mastadenovirus E* |  |
| DNA virus | *Epstein–Barr virus* |  |
| DNA virus | *Cytomegalovirus* |  |
| mycoplasma, chlamydia | *Chlamydia pneumoniae* |  |
| mycoplasma, chlamydia | *Chlamydia psittaci* |  |
| mycoplasma, chlamydia | *Chlamydia trachomatis* |  |
| mycoplasma, chlamydia | *Ureaplasma urealyticum* |  |
| mycoplasma, chlamydia | *Mycoplasma pneumoniae* |  |
| mycoplasma, chlamydia | *Mycoplasma hominis* |  |
| parasite | *Pentatrichomonas hominis* |  |
| parasite | *Leishmania* |  |
| parasite | *Plasmodium* |  |
| parasite | *Entamoeba histolytica* |  |
|  |  |  |
| **Drug Resistance Mutations** |  |  |
| **Anti-TB drugs** | **Resistant genes** | **Mutation sites** |
| Rifampicin | *rpoB* | D435Y |
| Rifampicin | *rpoB* | S450L |
| Rifampicin | *rpoB* | D435V |
| Rifampicin | *rpoB* | D435F |
| Rifampicin | *rpoB* | H445Y |
| Rifampicin | *rpoB* | H445D |
| Rifampicin | *rpoB* | H445L |
| Rifampicin | *rpoB* | H445R |
| Rifampicin | *rpoB* | H445N |
| Rifampicin | *rpoB* | H445C |
| Rifampicin | *rpoB* | H445S |
| Rifampicin | *rpoB* | S450W |
| Rifampicin | *rpoB* | S450F |
| Rifampicin | *rpoB* | L452P |
| Rifampicin | *rpoB* | L430P |
| Rifampicin | *rpoB* | Q432K |
| Rifampicin | *rpoB* | Q432P |
| Rifampicin | *rpoB* | Q432L |
| Rifampicin | *rpoB* | S441L |
| Rifampicin | *rpoB* | S441Q |
| Rifampicin | *rpoB* | 1296_ins_3_a_attc |
| Rifampicin | *rpoB* | 1328_ins_3_t_tgac |
| Isoniazid | *inhA* | c-777t |
| Isoniazid | *inhA* | a-778g |
| Isoniazid | *inhA* | t-8c |
| Isoniazid | *inhA* | t-8a |
| Isoniazid | *inhA* | t-8g |
| Isoniazid | *inhA* | S94A |
| Isoniazid | *inhA* | L203L |
| Isoniazid | *katG* | S315T |
| Isoniazid | *katG* | S315N |
| Isoniazid | *ahpC promoter region* | g-6a |
| Isoniazid | *ahpC promoter region* | c-10t |
| Isoniazid | *ahpC promoter region* | c-12t |
| Isoniazid | *ahpC promoter region* | c-30t |
| Pyrazinamide | *pncA* | 318_del_1_ga_g |
| Pyrazinamide | *pncA* | 395_del_9_ccgaccacat_c |
| Pyrazinamide | *pncA* | 465_ins_1_c_ca |
| Pyrazinamide | *pncA* | Q10P |
| Pyrazinamide | *pncA* | Q10R |
| Pyrazinamide | *pncA* | G97D |
| Pyrazinamide | *pncA* | G97C |
| Pyrazinamide | *pncA* | G97S |
| Pyrazinamide | *pncA* | G97R |
| Pyrazinamide | *pncA* | V7G |
| Pyrazinamide | *pncA* | V7L |
| Pyrazinamide | *pncA* | V7A |
| Pyrazinamide | *pncA* | C14R |
| Pyrazinamide | *pncA* | Q141* |
| Pyrazinamide | *pncA* | Q141P |
| Pyrazinamide | *pncA* | L4W |
| Pyrazinamide | *pncA* | L4S |
| Pyrazinamide | *pncA* | I133T |
| Pyrazinamide | *pncA* | T135P |
| Pyrazinamide | *pncA* | V139A |
| Pyrazinamide | *pncA* | V139G |
| Pyrazinamide | *pncA* | D8G |
| Pyrazinamide | *pncA* | D8N |
| Pyrazinamide | *pncA* | W68C |
| Pyrazinamide | *pncA* | W68G |
| Pyrazinamide | *pncA* | W68R |
| Pyrazinamide | *pncA* | Y103* |
| Pyrazinamide | *pncA* | Y103C |
| Pyrazinamide | *pncA* | Y103H |
| Pyrazinamide | *pncA* | T47A |
| Pyrazinamide | *pncA* | A134V |
| Pyrazinamide | *pncA* | I6T |
| Pyrazinamide | *pncA* | S67P |
| Pyrazinamide | *pncA* | R154G |
| Pyrazinamide | *pncA* | D12A |
| Pyrazinamide | *pncA* | D12G |
| Pyrazinamide | *pncA* | D12E |
| Pyrazinamide | *pncA* | G132S |
| Pyrazinamide | *pncA* | G132A |
| Pyrazinamide | *pncA* | L85P |
| Pyrazinamide | *pncA* | L85R |
| Pyrazinamide | *pncA* | T160P |
| Pyrazinamide | *pncA* | L182S |
| Pyrazinamide | *pncA* | V180F |
| Pyrazinamide | *pncA* | V180G |
| Pyrazinamide | *pncA* | P69L |
| Pyrazinamide | *pncA* | I90S |
| Pyrazinamide | *pncA* | L159R |
| Pyrazinamide | *pncA* | P62L |
| Pyrazinamide | *pncA* | A102P |
| Pyrazinamide | *pncA* | V155G |
| Pyrazinamide | *pncA* | D63A |
| Pyrazinamide | *pncA* | C138R |
| Pyrazinamide | *pncA* | G105V |
| Pyrazinamide | *pncA* | S164P |
| Pyrazinamide | *pncA* | T142A |
| Pyrazinamide | *pncA* | T142M |
| Ethambutol | *embB* | M306V |
| Ethambutol | *embB* | M306I |
| Ethambutol | *embB* | G406S |
| Ethambutol | *embB* | G406D |
| Ethambutol | *embB* | G406A |
| Ethambutol | *embB* | Q497K |
| Ethambutol | *embB* | Q497R |
| Ethambutol | *embB* | Y319S |
| Ethambutol | *embB* | Y319C |
| Ethambutol | *embB* | D328Y |
| Ethambutol | *embB* | E368A |
| Ethambutol | *embA* | c-12t |
| Ethambutol | *embA* | c-16t |
| Ethambutol | *embA* | c-16g |
| Streptomycin | *rpsL* | K43R |
| Streptomycin | *rpsL* | K88R |
| Streptomycin | *rrs* | a514c |
| Streptomycin | *rrs* | a514t |
| Streptomycin | *rrs* | C517T |
| Streptomycin | *gid* | 103_del_1_gc_g |
| Streptomycin | *gid* | 352_del_1_gc_g |
| Streptomycin | *gid* | Q125* |
| Streptomycin | *gid* | G69D |
| Streptomycin | *gid* | P75R |
| Levofloxacin/Moxifloxacin | *gyrA* | D94G |
| Levofloxacin/Moxifloxacin | *gyrA* | D94N |
| Levofloxacin/Moxifloxacin | *gyrA* | D94H |
| Levofloxacin/Moxifloxacin | *gyrA* | D94Y |
| Levofloxacin/Moxifloxacin | *gyrA* | D94A |
| Levofloxacin/Moxifloxacin | *gyrA* | A90V |
| Levofloxacin/Moxifloxacin | *gyrA* | S91P |
| Levofloxacin/Moxifloxacin | *gyrA* | D89G |
| Levofloxacin/Moxifloxacin | *gyrA* | G88C |
| Levofloxacin/Moxifloxacin | *gyrB* | E501D |
| Levofloxacin/Moxifloxacin | *gyrB* | A504V |
| Linezolid | *rplC* | C154R |
| Bedaquinoline | *atpE* | D28V |
| Bedaquinoline | *atpE* | D28P |
| Bedaquinoline | *atpE* | D28G |
| Bedaquinoline | *atpE* | D28N |
| Bedaquinoline | *atpE* | E61D |
| Bedaquinoline | *atpE* | A63P |
| Bedaquinoline | *atpE* | A63V |
| Bedaquinoline | *atpE* | L59V |
| Bedaquinoline | *RV0678* | M1A |
| Cycloserine | *alr* | D344N |
| Cycloserine | *alr* | S261N |
| Clofazimine | *RV0678* | 192_ins_1_g_gg |
| Clofazimine | *RV0678* | 192_del_1_cg_c |
| Ethyl thioisamide/ Propyl thioisamide | *inhA* | c-15t |
| Ethyl thioisamide/ Propyl thioisamide | *inhA* | a-16g |
| Ethyl thioisamide/ Propyl thioisamide | *inhA* | t-8a |
| Ethyl thioisamide/ Propyl thioisamide | *inhA* | t-8c |
| Ethyl thioisamide/ Propyl thioisamide | *ethA* | 111_del_1_ct_c |
| Ethyl thioisamide/ Propyl thioisamide | *ethA* | R207G |
| Ethyl thioisamide/ Propyl thioisamide | *ethA* | M1R |
| Para-aminosalicylic acid | *folC* | E40G |
| Para-aminosalicylic acid | *folC* | E40K |
| Para-aminosalicylic acid | *folC* | E40Q |
| Para-aminosalicylic acid | *folC* | E40R |
| Para-aminosalicylic acid | *folC* | I43A |
| Para-aminosalicylic acid | *folC* | I43T |
| Para-aminosalicylic acid | *thyA* | R235P |
| Para-aminosalicylic acid | *thyA* | R235L |
| Para-aminosalicylic acid | *thyA* | H147R |
| Dramani | *ddn* | L49P |

**Reference**

1. Chen S, Zhou Y, Chen Y, Gu J. fastp: an ultra-fast all-in-one FASTQ preprocessor. Bioinformatics 2018. 34:i884–90. Available from: http://dx.doi.org/10.1093/bioinformatics/bty560

2. Nurk S, Koren S, Rhie A, Rautiainen M, Bzikadze AV, Mikheenko A, et al. The complete sequence of a human genome. Science 2022. 376:44–53. Available from: http://dx.doi.org/10.1126/science.abj6987

3. Jung Y, Han D. BWA-MEME: BWA-MEM emulated with a machine learning approach. Bioinformatics 2022. 38:2404–13. Available from: http://dx.doi.org/10.1093/bioinformatics/btac137

4. Chitale P, Lemenze AD, Fogarty EC, Shah A, Grady C, Odom-Mabey AR, et al. A comprehensive update to the Mycobacterium tuberculosis H37Rv reference genome. Nat Commun 2022. 13:7068. Available from: http://dx.doi.org/10.1038/s41467-022-34853-x
